# Evaluation of physical fitness and health of young children aged between 3 and 6 based on cluster and factor analyses

**DOI:** 10.1186/s12889-024-17660-5

**Published:** 2024-02-09

**Authors:** Rui Zhao, Xue Li, Junwei Wang, Lanchuan Zhang, Zhanle Gao

**Affiliations:** School of Sports Medicine and Health, Chengdu Sport University, #2, Tiyuan Road, Wuhou District, Chengdu, 610041 China

**Keywords:** Preschool children, Physical fitness, Cluster analysis, Factor analysis

## Abstract

**Background:**

As life improves and sedentary time increases, young children's physical fitness gradually declines.

**Methods:**

Multi-stage stratified whole cluster sampling was utilized to sample 5584 preschoolers. Young infants' morphology, function, and quality were revealed using cluster and factor analysis.

**Results:**

The cluster analysis separated 3–6-year-olds into two genders: 1,551 men in group A "high physical fitness" 1,499 men in group B "low physical fitness"; 1,213 women in group A and 1,321 women in group B. Young children's fitness was measured by standing long jump(1.00), weight(1.00), and height(1.00). A cluster analysis of 3–4-year-olds classified them into three groups: 272 “muscular strength,” 75 “average physical fitness,” and 250 “low agility.” Young children's health depends on weight (1.00), height (0.57), and chest circumference (0.54). A cluster analysis of the 4–5-year-olds classified them into two groups: 1070 “balance” and 806 “muscular strength.” Young children’s health depends on weight (1.00), height (0.74), and chest circumference (0.71). A cluster analysis of the 5–6-year-olds divided them into three groups: 1762 “high physical fitness,” 384 “obese,” and 105 “low physical fitness.” Young children’s physical health depends on BMI (1.00), weight (1.00), and chest circumference (1.00). Factor analysis demonstrated that muscle strength, body shape, cardiovascular variables, and physical fitness composite components affected young children's health.

**Conclusion:**

Women should focus on motor function and strength, while men on flexibility. Male group B “low physical fitness” should focus on strength, motor function, and balance, whereas male group A “high physical fitness” should focus on flexibility. Then, female group A “high physical fitness” should emphasize variety.2) For 3–4-year-olds, group A “muscular strength” should focus on flexibility, and group C “low agility” on motor function. 3) For 4–5-year-olds, group A “balanced” should focus on strength and motor function; 4) For 5–6-year-olds, group B “obese” should emphasize weight loss, and group C “low fitness” should emphasize strength, motor function, and flexibility; 5) Young children’s physical fitness depends on muscle strength, body shape, cardiovascular factors, and physical fitness composite.

**Supplementary Information:**

The online version contains supplementary material available at 10.1186/s12889-024-17660-5.

## Introduction

The physical fitness of children determines the health of the general population and the future prosperity of the nation [1]. Children between the ages of 3 and 6 years old show a declining trend in their level of physical fitness, according to numerous studies [2–6]. The prolonged immunization of young children since the new coronavirus pneumonia epidemic has made this trend more pronounced. Early childhood is crucial for encouraging and establishing beneficial health behaviors from the lifelong development perspective. Early childhood physical fitness is closely related to adult physical fitness.

Building a great scientific approach for monitoring physical fitness and health is especially crucial given the inadequate level of young children’s physical fitness and health [7, 8]. Physical fitness for children and adolescents is vital to a nation’s ability to compete internationally. The American “FITNESSGRAM” (children and adolescents physical health assessment system) [9], the Australian “Fit-4-Fun test battery” [10], the European “EUROFIT test battery” [11, 12], the Japanese “New Physical Fitness Test Implementation Guidelines” [13], and other Western nations have created their physical fitness testing standards for children and adolescents, including the Canadian “Physical Literacy Assessment for Children and Youth” [14], Already used internationally [15, 16]. In 2003, China released the Physical Literacy Assessment for Youth [13] and the first National Physical Fitness Standard (Early Childhood Section), also known as the 2003 Standard. Despite the enthusiasm of researchers about preschoolers' interest in physical fitness, there are few international physical fitness assessments for preschoolers. Even some countries still do not have official physical fitness testing methods for preschoolers [17]. Conducting an accurate, personalized assessment is currently impossible because physical fitness evaluations domestically and internationally are based on age segments. In addition, the overall score of children’s physical fitness tests is evaluated comprehensively without considering individual differences within the same age group or the issue of mutation among adjacent grades.

Grading physical fitness and measuring variances using defined criteria are insufficient to reflect an individual’s physical fitness because young children’s physical development varies significantly at different stages. One of the big data–based unsupervised machine learning techniques is cluster analysis. Clustering is generally used to investigate students’ physical fitness and health. Clustering is a technique for locating a cluster structure in a dataset characterized by maximum similarity within the same cluster and maximum dissimilarity among different groups [14, 18]. The contribution rate of the clustering structure can also indicate the significance of particular indicators to the data. Based on this, this study uses cluster analysis to examine the physical well-being of young children, assess the level of material well-being in children aged 3–6 years old, and develop the physical quality evaluation standard for children aged 3–6 years old.

## Methods

### Participants and ethics statement

In this study, we chose eight cities and four suburban counties in Sichuan Province using multi-stage stratified cluster sampling. We then decided on two public kindergartens in each town and one public kindergarten in each suburban county. Finally, 5584 children were randomly chosen from the public kindergartens sampled. The algorithm of full age was applied; thus, the survey date was deducted from the respondents’ date of birth. Those whose date of birth was reported using the lunar calendar had their date converted to the solar calendar, and their calculations were then made with a two-decimal place precision. For this study, young children ages 3–6 years old with complete height and weight records were chosen. The following criteria must be met to include a kid: (1) enrollment in the school with informed parental and school agreement; (2) voluntary participation in the physical fitness test; (3) in good health with no history of infections or heart or lung conditions. Abnormal physical development, disability, or deformity is prohibited. All participants' guardians agreed to participate in this study and we obtained their informed consent. The Chengdu University of Physical Education and Sports’ Ethics Committee examined this work (ID: [2022]59). The study also complied with the latestversion of the Helsinki Declaration.

## Methods

### Test method

Physical fitness tests are conducted concerning the guidelines in the National Physical Fitness Monitoring Workbook (Early Childhood Section) (hereafter referred to as the “Standard”) [19]. Since the promulgation of this document, surveys have been conducted among a large number of children between the ages of 3 and 6, which are able to objectively reflect the real-time physical health status of children in China with a high degree of reliability and validity. The equipment chosen by China’s General Administration of Sport is the physical fitness test device (Jianmin brand). The test will be administered in September of 2022 and includes eight indicators of height, weight, seated forward bending, standing long jump, 10-m shuttle run, tennis throw, double-legged jumps, balance beam, chest circumference, body mass index (BMI), heart rate, systolic blood pressure, diastolic blood pressure, 20-m run, and one-leg stance. Based on the Standard, these additional indicators have also been added. For a complete list of measurements, see the overview of the original data in the attached file. This overview includes measurement metrics and specific values.

### Additional indicators

Chest Circumference: The testing tool is a soft tape measure. In the seated position, the person taking the measurement should stand on the right side or in front of the child, with the beginning of the tape measure placed under the nipple on the right side of the subject, the right hand holding the tape measure around the back, through the lower edge of the shoulder blades bilaterally and then measure the left side of the thorax, through the left side of the nipple under the tape measure and back to the zero point of the soft tape measure again. The average of the values between the two breaths is usually taken, and the value against which the tape is returned to the zero point is the chest circumference.

BMI: Reflects the relationship between body weight and height, and has been used by WHO as an important indicator of body fatness and thinness.BMI = Weight (kg) ÷ Height^^2^ (m).

Heart rate: Utilize the Huawei smart bracelet. In a quiet state, wear the bracelet at the wrist, light up the screen, and press the touch button. After pressing, adjust the menu of Huawei bracelet so that it is adjusted to the heart rate test menu, at this time, stand still and wait for the results of the measurement, after the measurement is finished, the heart rate will be displayed on the screen.

Systolic/diastolic blood pressure: The test equipment is a commonly used column-type mercury sphygmomanometer and a medical stethoscope. The subject is seated with the right arm naturally stretched out in front, flat on the table, palm up. The "0" position of the sphygmomanometer should be at the same level as the subject's heart and right arm cuff. When the tester ties the cuff, it should be flat and loose, and the elbow socket should be fully exposed. Feel the position of the brachial artery and place the stethoscope stethoscope head on it so that the head is in close contact with the skin, but should not be pressed hard and tightly or tucked under the cuff. The stethoscope is inflated into the band so that the mercury column rises rapidly until the brachial artery pulsation is not heard and then rises by 20–30 mm Hg. Then the stethoscope is slowly deflated, and when the first pulse sound is heard, the height of the mercury column is systolic blood pressure; it continues to be deflated, and the pulse sound undergoes a series of changes, and the value of the height of the column at the moment of the disappearance of the pulse sound is diastolic blood pressure. The blood pressure test is designed to be heard at once, otherwise, it is re-measured. Record systolic and diastolic blood pressure in mmHg (mmHg = 0.1333 kPa).

20-m run: a test using a stopwatch. A 20-m-long, 1.22-m-wide straight track is drawn on a flat surface. During the test, at least two groups of subjects to stand in a standing starting position in front of the starting line, when you hear the "run" command, run to the end of the full force, the tester depending on the subject to start to open the meter timing. When the chest reaches the vertical plane of the starting line, the tester stops the watch.

Standing on one foot: A non-slip mat and stopwatch are used. For the test, the subject stands on the ground with feet naturally apart. One leg was then bent and lifted off the ground with eyes open. The attempt was repeated once for each leg and the time spent holding the position was noted.2. Cluster analysis.

Physical fitness indicators were grouped using a two-stage clustering strategy, one of the best ways to deal with massive data and automatically calculate the correct number of clusters. (The principle of cluster analysis is that individuals in the same category have greater similarity and individuals in different categories have greater differences.)Distances were clustered using the log-likelihood approach, and the clustering criterion utilized was the Bayes’ information criterion (BIC) [20]. The algorithm automatically determines the number of clusters using the ideal ratio of low BIC, high BIC variability, and high distance measurements. The quality of the clustering improves as the contour coefficient approaches 1. Each measure has a 0–1 importance scale, with values closer to 1 indicating substantially high importance. Two stages are involved in calculating the two-stage cluster analysis; the first stage analyzes the initial records and builds a feature tree of categories. The size of the Akaike information criterion (AIC) or (BIC) value and the fluctuation of the shortest distance between classes are used to determine the optimum number of categories in the second stage of systematic clustering using the feature tree. The two-stage clustering method uses the log-likelihood as a distance measure to handle continuous and categorical data. In addition, the amount of drop indicates the distance between two classes, j and s, in the log-likelihood, after they are combined [21]:

included among these, $$\begin{array}{c}{d}_{j,s}={\xi }_{j}+{\xi }_{s}-{\xi }_{\left(j,s\right)}\\ {\xi }_{V}={N}_{v}\left({\sum }_{l=1}^{{K}_{A}}\frac{1}{2}{\text{log}}\left(\widehat{\sigma }+{\widehat{\sigma }}_{vk}^{2}\right)+{\sum }_{k=1}^{{K}_{B}}{E}_{vk}\right)\\ {E}_{rk}=-\sum_{l=1}^{Lk}\frac{{N}_{vkl}}{{N}_{v}}log\frac{{N}_{rkl}}{{N}_{v}}\end{array}$$

where KA-number is the continuous variables used, KB-number is the categorical variables, Lk-number is the categories of the k categorical variable, Nv-number is the samples of category v, Nvkl-number is the pieces belonging to category v in which the k categorical variable takes the value of class l, is the estimate of the variance of the k continuous variable, (j,s) denotes that this new class is merged from categories j and s, and V represents either category j or s.

### Statistic analysis

Excel and SPSS26 software were used to enter and analyze mathematical statistics. Different items were grouped into cluster nodes based on the data of 15 indicators for assessing physical fitness. Moreover, cluster analysis was then carried out to determine the ideal number of clusters automatically. The data were described by mean and standard deviation (M ± SD), and a comparison among groups was conducted using the t-test or analysis of variance (ANOVA), with a test level of 0.05.

## Results

### Basic characteristics

The samples were divided into three age groups, with 3111, 1876, and 597 kids in each age group (Table 1). A total of 15 measurements, namely, height, weight, chest circumference, BMI, heart rate, systolic blood pressure, diastolic blood pressure, 10-m shuttle run, 20-m run, tennis throw, standing long jump, one-leg stance, and seated forward bending, were statistically significant (*P* < 0.05) in the analysis of the differences among the different gender groups, as shown in Table 2. Double-legged jumps and balance beam were not statistically significant (*P* > 0.05).

**Table 1:** Basic information on young children

**Age**		**Height(cm)**	**Weight(kg)**	**Total**
3–4		101.32 ± 3.87	16.08 ± 1.85	597
4–5		105.69 ± 4.50	17.39 ± 2.28	1876
5–6		115.70 ± 5.43	20.39 ± 3.36	3111
				5584

**Table 2:** Comparison of physical health scores of young children of different genders

Basic indicator	Male	Female	T	*P*
Height(cm)	111.32 ± 7.36	110.16 ± 7.67^**^	5.78	0.00
Weight(kg)	19.53 ± 3.57	18.84 ± 3.36^**^	7.46	0.00
Chest circumference(cm)	55.51 ± 3.62	54.09 ± 3.47^**^	14.95	0.00
BMI(kg.m^2^)	15.66 ± 1.57	15.44 ± 1.52^**^	5.26	0.00
Heart rate(b/min)	94.70 ± 14.87	96.52 ± 15.18^**^	-4.53	0.00
Systolic pressure(mmHg)	102.27 ± 11.40	99.33 ± 11.16^**^	9.81	0.00
Diastolic pressure(mmHg)	63.11 ± 9.45	61.14 ± 9.21^**^	7.85	0.00
10-m shuttle run(s)	7.02 ± 1.01	7.28 ± 1.06^**^	-9.22	0.00
20-m run(s)	5.55 ± 0.86	5.71 ± 0.86^**^	-7.05	0.00
Tennis throw(m)	5.76 ± 2.32	4.60 ± 1.67^**^	21.75	0.00
Double-legged jumps(s)	6.59 ± 3.22	6.61 ± 3.65	-0.23	0.82
Standing for jump(cm)	92.59 ± 21.46	87.44 ± 19.73^**^	9.34	0.00
Balance beam(s)	20.95 ± 22.66	21.81 ± 23.97	-1.37	0.17
One-leg stance(s)	24.73 ± 28.97	41.17 ± 46.74^**^	-15.42	0.00
Seated forward bending(cm)	7.94 ± 4.59	12.14 ± 4.37^**^	-34.72	0.00

* = Significant change *p*-value < 0.05 from pre-evaluation

** = Significant change *p*-value < 0.01 from pre-evaluation

### Cluster Distribution

#### Cluster analysis by gender

The information criterion for cluster analysis identified two ideal clusters, and the clusters’ quality was satisfactory. The cluster analysis’s findings (Table 3) revealed that males were split into two groups, with 1551 falling into category A “high physical fitness” and 1499 falling into category B “low physical fitness.” Two classes of females were created, with 1213 falling into the “high physical fitness type” category and 1321 falling into the “low physical fitness type” category. The findings of the ANOVA demonstrated that the disparities among the various classifications, except heart rate, were statistically significant (*P* < 0.05), regardless of gender. All physical fitness indicators for males in category A were superior to those in category B, except for seated forward bending; all physical fitness indications for females in category B were superior to those in category A. The summary of the clustering model revealed that standing for jump (1.00), weight (1.00), and height (1.00) were the top 3 contributors to the category structure of young children’s physical fitness test results.

**Table 3:** Physical health clustering of young children of different genders

**Basic indicator**	**Male**				**Female**			
	A	B	*T*	*P*	A	B	*T*	*P*
	(*n* = 1551)	(*n* = 1499)			(*n* = 1213)	(*n* = 1321)		
Height(cm)	116.65 ± 5.12	105.83 ± 4.82^**^	60.05	0.00	103.90 ± 4.49	115.90 ± 5.05^**^	-63.00	0.00
Weight(kg)	21.81 ± 3.29	17.19 ± 1.96^**^	46.91	0.00	16.53 ± 1.79	20.96 ± 3.06^**^	-43.98	0.00
Chest circumference(cm)	57.54 ± 3.52	53.41 ± 2.28^**^	38.32	0.00	52.19 ± 2.28	55.82 ± 3.47^**^	-30.83	0.00
BMI(kg.m2)	15.98 ± 1.79	15.33 ± 1.23^**^	11.65	0.00	15.30 ± 1.20	15.57 ± 1.76^**^	-4.47	0.00
Heart rate(b/min)	94.96 ± 13.81	94.43 ± 15.90	0.98	0.33	97.04 ± 16.06	96.03 ± 14.30	1.67	0.09
Systolic pressure(mmHg)	105.08 ± 10.47	99.37 ± 11.08^**^	14.63	0.00	96.93 ± 11.37	101.54 ± 10.49^**^	-10.62	0.00
Diastolic pressure(mmHg)	65.15 ± 9.06	61.02 ± 9.38^**^	12.37	0.00	59.58 ± 9.63	62.58 ± 8.57^**^	-8.30	0.00
10-m shuttle run(s)	6.47 ± 0.56	7.58 ± 1.06^**^	-36.33	0.00	7.99 ± 1.03	6.62 ± 0.54^**^	42.41	0.00
20-m run(s)	5.08 ± 0.52	6.03 ± 0.88^**^	-36.44	0.00	6.29 ± 0.80	5.17 ± 0.49^**^	42.87	0.00
Tennis throw(m)	7.13 ± 2.21	4.35 ± 1.41^**^	41.26	0.00	3.48 ± 1.00	5.62 ± 1.49^**^	-42.07	0.00
Double-legged jumps(s)	5.34 ± 1.34	7.88 ± 3.98^**^	-23.78	0.00	7.98 ± 4.77	5.35 ± 1.19^**^	19.39	0.00
Standing for jump(cm)	106.01 ± 16.28	78.74 ± 16.83^**^	45.49	0.00	73.72 ± 13.99	100.00 ± 15.34^**^	-44.93	0.00
Balance beam(s)	11.57 ± 10.12	30.65 ± 27.50^**^	-25.59	0.00	32.77 ± 29.72	11.74 ± 8.98^**^	24.53	0.00
One-leg stance(s)	37.10 ± 34.92	11.95 ± 11.16^**^	26.60	0.00	16.66 ± 17.14	63.70 ± 53.52^**^	-29.26	0.00
Seated forward bending(cm)	7.73 ± 4.66	8.17 ± 4.52^*^	-2.65	0.01	11.59 ± 4.10	12.65 ± 4.54^**^	-6.15	0.00

* = Significant change *p*-value < 0.05 from pre-evaluation

** = Significant change *p*-value < 0.01 from pre-evaluation

### Cluster Analysis by age group

1. Cluster analysis of 3–4-year-old children.

**Fig. 1:**
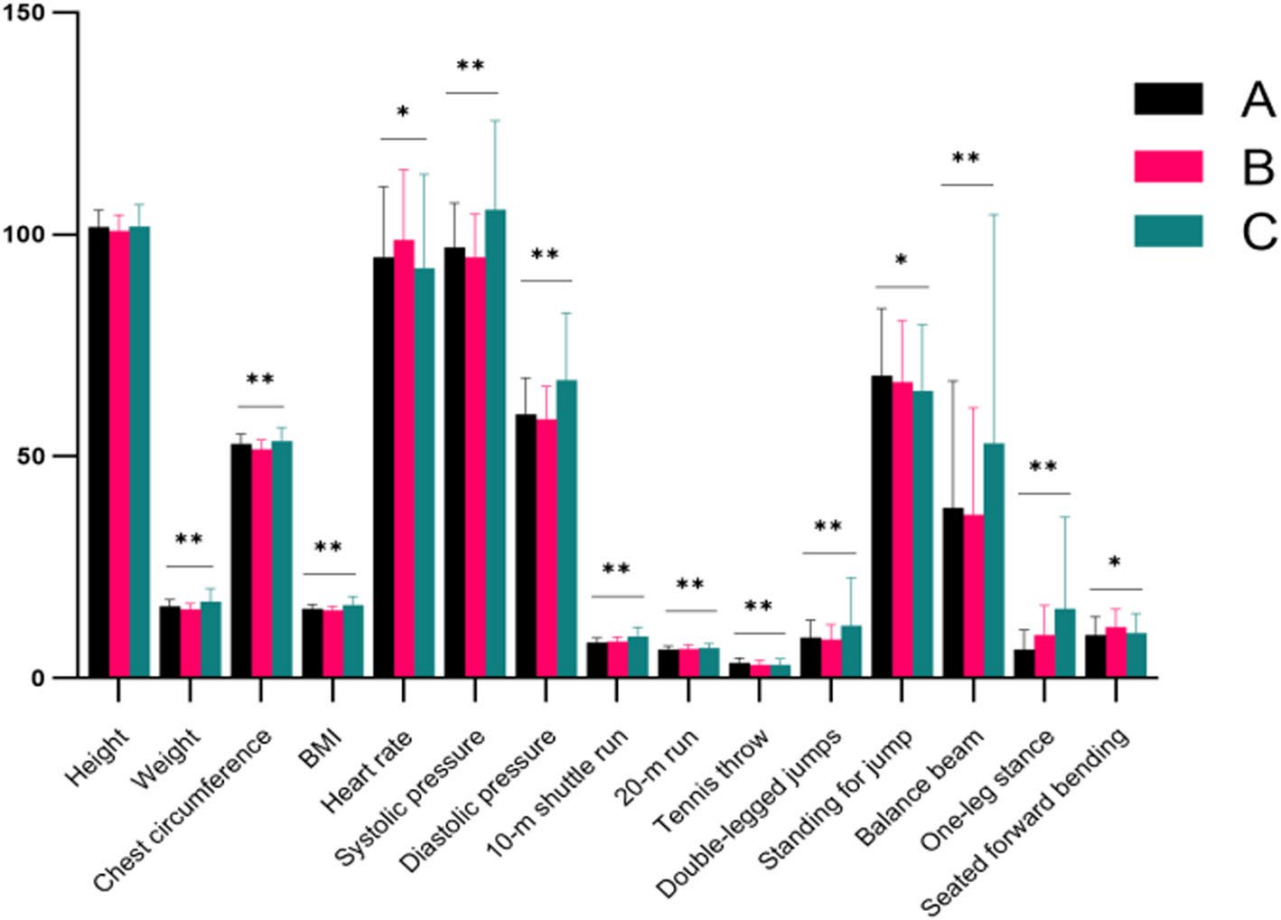
Cluster analysis of physical fitness scores of the 3–4-year-olds. Notes: **A** (*n* = 272):muscular strength, **B** (*n* = 75):average physical fitness, **C** (*n* = 250):low agility. * = Significant change *p*-value < 0.05 from pre-evaluation. ** = Significant change *p*-value < 0.01 from pre-evaluation

The outcomes of the cluster analysis were nicely clustered into three categories(Fig. 1). Group C “low agility” has 250 children, group A “muscular strength” has 272 children, and group B “average physical fitness” has 75 children. The ANOVA findings revealed that, except for height, statistically significant differences exist in comparisons of all indicators among the three categories (*P* < 0.05). Group A exhibited great explosive power, superior motor function, and upper solid limb, waist, and abdominal strength. Group B showed average overall physical fitness, whereas group C exhibited a high BMI, decent physical nutrition, and weak motor function. Weight (1.00), height (0.57), and chest circumference (0.54) were the top 3 contributors to the categorical structure affecting the physical fitness test scores of young children, according to a summary of the clustering model.

(2) Cluster analysis of the 4–5-year-olds.

**Fig. 2:**
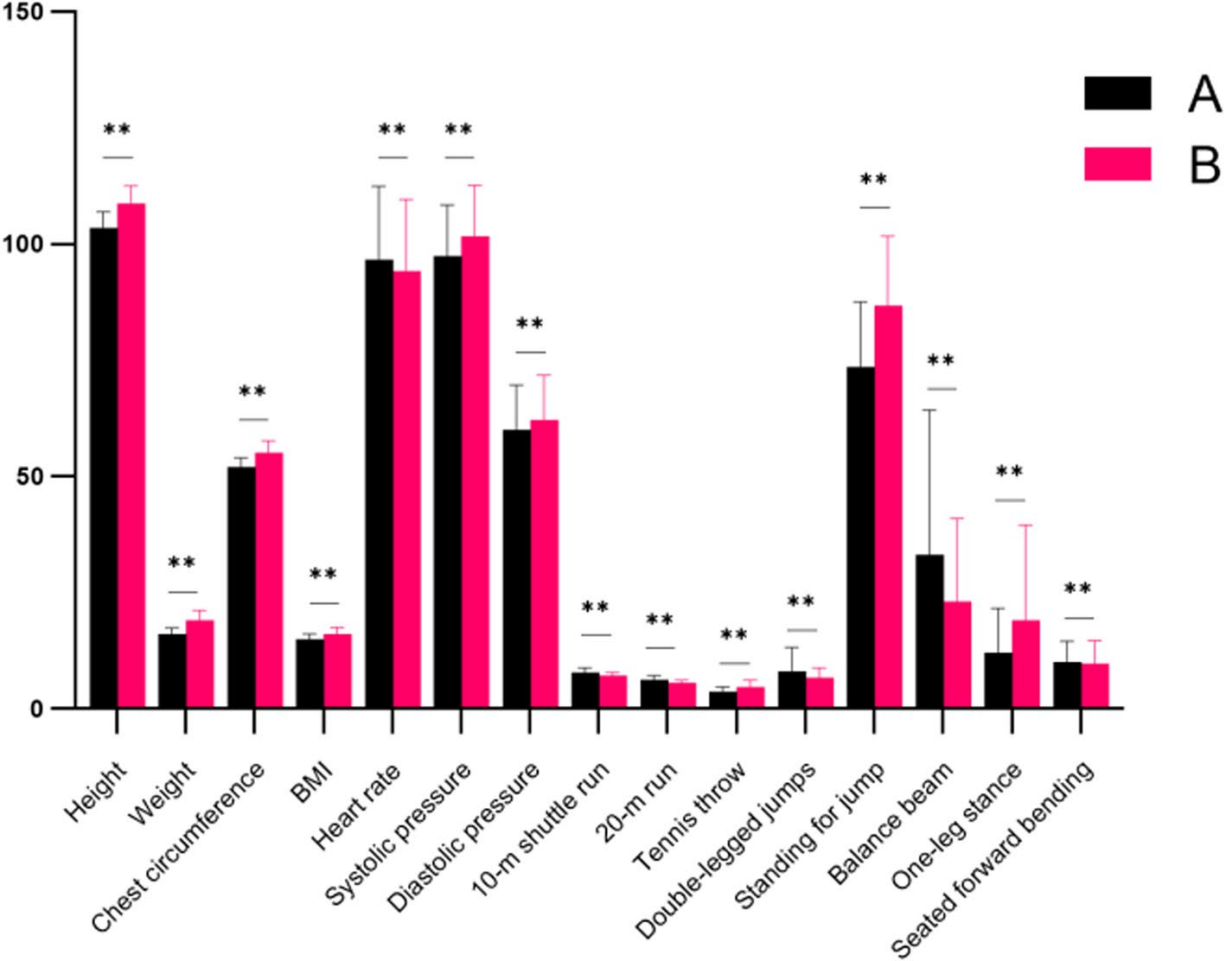
Cluster analysis of physical fitness scores of the 4–5-year-olds. Notes: **A** (*n* = 1070):balance, **B** (*n* = 806):muscle strength. * = Significant change *p*-value < 0.05 from pre-evaluation. ** = Significant change *p*-value < 0.01 from pre-evaluation

The cluster analysis findings were divided into two categories with good clustering(Fig. 2), 1070 in group A “balance” and 806 in group B “muscle strength.” The differences between the two categories were statistically significant (*P* < 0.01) according to the t-test results, except for seated forward bending. The heart rate was higher in group A, and physical nutrition, motor function, and muscle strength were higher in group B. The top 3 parameters that contributed to the category structure of young children’s physical fitness test scores were weight (1.00), height (0.74), and chest circumference (0.71), according to a summary of the clustering model.

(3) Cluster analysis of children aged 5–6 years old.

**Fig. 3:**
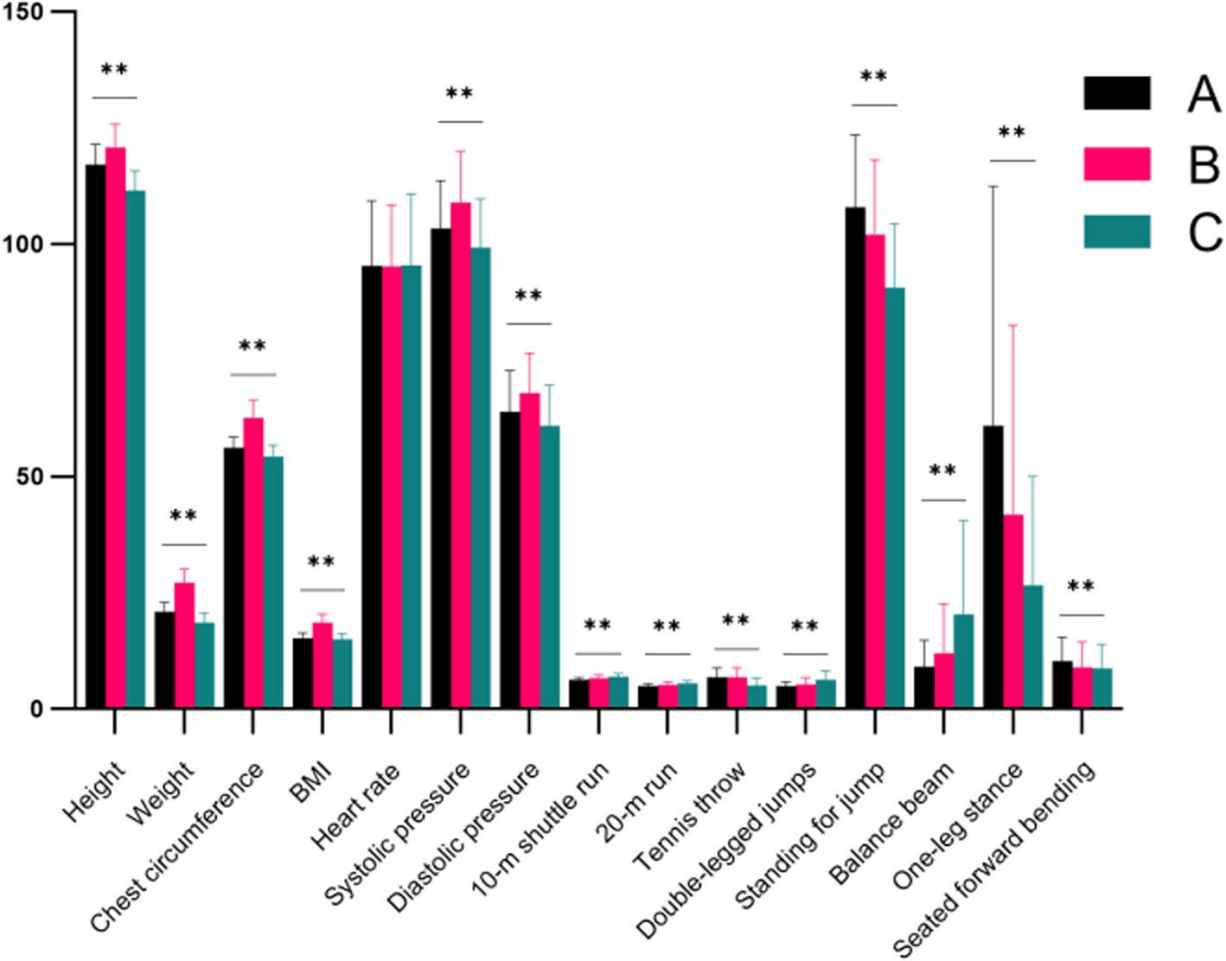
Cluster analysis of physical fitness scores of the 5–6-year-olds. Notes: **A** (*n* = 1762):high physical fitness, **B** (*n* = 384):obese, **C** (*n* = 1055):low physical fitness. * = Significant change *p*-value < 0.05 from pre-evaluation. ** = Significant change *p*-value < 0.01 from pre-evaluation

Three categories were created from the cluster analysis results, and the clustering was effective(Fig. 3). Group A “high physical fitness” has 1762, group B “obese” has 384, and group C “low physical fitness” has 1055. The ANOVA results showed that the differences in comparing all indicators except for heart rate among different categories were statistically significant (*P* < 0.01). Among them, group A has vital motor function, muscle strength, and flexibility; group B has an important BMI, and all other indicators of physical fitness are good; and group C is thin and has weak motor function, muscle strength, and flexibility. A summary of the clustering model revealed that BMI (1.00), weight (1.00), and chest circumference (1.00) were the top 3 contributors to the categorical structure affecting the physical fitness test scores of young children.

### Factor analysis

#### Factor analysis applicability test

The data are highly correlated and acceptable for factor analysis, as shown by the KMO value of 0.743 (> 0.5) and Bartlett’s test of sphericity value of 0.00 (*P* < 0.05).

### Extraction of the public factor for the comprehensive evaluation of physical fitness

According to the eigenvalue more significant than one principle, four fundamental elements may be retrieved from the scores of the following 15 variables using the SPSS software (Table 4). These four components together account for 63.88% of the total variance, which implies that they can explain 63.88% of the data from the initial 15 indicators (Table 5). The loading matrix for the four fundamental elements was constructed following the rotation procedure using the maximum variance approach (Table 6). The correlation coefficient between each original variable and the corresponding factor, which shows the level of correlation between the original variable and related element, determines the statistical significance of the factor loading values in the table.

**Table 4:** Factor analysis eigenvalues

Item	Total	Factor	Variance	Variance %	Cumulative %
X1	5.10	1	5.10	34.03	34.03
X2	1.99	2	1.99	13.24	47.26
X3	1.38	3	1.38	9.22	56.49
X4	1.11	4	1.11	7.39	63.88
X5	0.95	5	0.95	6.31	70.19
X6	0.78	6	0.78	5.21	75.40
X7	0.70	7	0.70	4.69	80.09
X8	0.68	8	0.68	4.56	84.65
X9	0.52	9	0.52	3.50	88.15
X10	0.45	10	0.45	3.02	91.17
X11	0.39	11	0.39	2.60	93.77
X12	0.37	12	0.37	2.43	96.20
X13	0.33	13	0.33	2.20	98.40
X14	0.24	14	0.24	1.58	99.98
X15	0.00	15	0.00	0.03	100

Total: The degree of contribution of each factor before the rotation of the indicator. The sum of this value is matched to the number of items, with larger values representing greater factor contribution.

Variance %: It is mainly used to determine how many factors are appropriate to extract. The larger the variance explained indicates that the factor contains more information about the original data.

Cumulative %: The idea is to look at the contribution of the factors to the explanation of the variable (which can be interpreted as how many factors are actually needed to express the variable as 100%).

**Table 5:** Contribution of the four factors to the original data

Item	Extraction	Factor	Variance	Variance %	Cumulative %
X1	0.76	1.00	5.10	34.03	34.03
X2	0.94	2.00	1.99	13.24	47.26
X3	0.82	3.00	1.38	9.22	56.49
X4	0.82	4.00	1.11	7.39	63.88

**Table 6:** Factor loading matrix

Item	1	2	3	4
Height(cm)	0.818	0.104	0.040	-0.005
Weight(kg)	-0.778	-0.104	-0.073	0.037
Chest circumference(cm)	-0.769	-0.120	-0.063	0.040
BMI(kg.m2)	0.737	0.446	0.099	-0.074
Heart rate(b/min)	0.707	0.192	0.083	-0.166
Systolic pressure(mmHg)	-0.598	-0.005	0.010	-0.087
Diastolic pressure(mmHg)	-0.564	0.021	-0.065	-0.057
10-m shuttle run(s)	0.531	0.097	-0.036	0.399
20-m run(s)	-0.174	0.882	0.092	0.039
Tennis throw(m)	0.447	0.852	0.126	-0.035
Double-legged jumps(s)	0.337	0.825	0.126	-0.082
Standing for jump(cm)	0.080	0.121	0.889	0.023
Balance beam(s)	0.121	0.174	0.864	-0.006
One-leg stance(s)	-0.007	0.015	-0.112	0.868
Seated forward bending(cm)	-0.035	-0.096	0.279	0.399

The results of the 15 indices can be categorized and analyzed using cluster analysis. However, factor analysis examined the relationships and influencing elements that shape young children’s physical fitness. (Factor analysis is a method of dimensionality reduction by converting multiple variables into a few, thus reducing the complexity of the problem analysis.) The combined significance of several indices with high weights in each linear combination formula was used to determine the importance of the first four principal components. The contribution rates revealed that the four main components’ cumulative contribution rates were 34.03%, 47.26%, 56.49%, and 63.88%, respectively (Table 4). The first factor, which was standardized, had significant loadings for height, tennis throw, and standing long jump as muscle strength factors; the second factor, which was normalized, had substantial loadings for weight, chest circumference, and BMI as body shape factors; the third factor, which was standardized, had significant loadings for systolic and diastolic blood pressure in young children as cardiovascular factors; and the fourth factor has significant loadings on heart rate, one leg stance, and sit and reach, mainly reflecting physiological function, balance function, and flexibility function, and is a composite factor of physical fitness.

## Discussion

Individual growth and development in early childhood are highly differentiated, and individuals have different rates and levels of growth and expansion at other times. As a result, using the same set of standards to assess young children’s physical fitness may not have an accurate reflection of how young children find themselves in terms of their physical fitness. Cluster analysis divides young children’s physical fitness into several categories, which may reveal the variations between types. The cluster model framework can also highlight the significance of specific biological fitness indicators for physical health [22]. Cluster analysis, a technique for assessing physical fitness, is focused, objective, easy to use, and effective for assessing young children’s physical fitness [23].

According to the rule of early children’s physical fitness development, young children’s body flexibility and quiet heart rate decline with age, whereas their tennis throw, standing for jump, 10-m shuttle run, and 20-m run increase. The results of this study show that male toddlers perform better than female toddlers in terms of height, weight, chest circumference, BMI, systolic pressure, diastolic pressure, 10-m shuttle run, 20-m run, tennis throw, and standing for jump [24]. However, they perform worse than female toddlers in terms of seated forward bending, one-leg stance, and heart rate. Similar to the findings of Cristina Cadenas-Sanchez et al. [25], gender disparities in several early childhood measures of physical fitness have emerged [26]. Male toddlers prefer daily sports that emphasize attributes, such as strength, speed, and agility, and have low levels of adiposity [27, 28], which may have neglected the stretching of muscles, tendons, and ligaments, including developmental patterns and children’s pure application of movements. This case is primarily related to growth skills. Flexibility exercises should be emphasized in toddler boys, and activities should be strengthened in toddler girls to develop well-rounded physical fitness development. As shown in earlier studies, a negative connection exists between physical activity and resting heart rate [29, 30]. Physical activity also boosts parasympathetic nerves while decreasing sympathetic activity at rest [31]. Male toddlers’ heart rates are often lower than those of female toddlers, which may be related to the fact that they are physically active and like participating in physical activities. Similar to the findings of Zeng Qiang et al. [32], this study revealed no difference in the ability of male and balance beam for female toddlers and double-legged jumps. Less variation exists between male and female toddlers as they become older when doing the double-legged jumps and walking the balance beam, with the age range between 3 and 4 years as the most variable [33].

According to the study’s findings, no discernible difference exists in heart rate between males and females. This result may indicate that this age group is not particularly sensitive to differences in heart rate between males and females. Children’s and teenagers’ resting heart rates correlate highly with adult cardiovascular development [34]. In contrast to a low resting heart rate, a high resting heart rate was linked to a high risk of all-cause death, according to a recent meta-analysis [35]. Therefore, young children need to monitor their resting heart rates. The results of this study suggest that the male group B “low physical fitness” should strengthen all physical fitness exercises, the male group A “high physical fitness” should strengthen flexibility exercises, and the female group A “high physical fitness” should strengthen all physical fitness exercises. The “high physical fitness” female group should improve their physical fitness exercises.

In the age range of 3–4 years old, group A “muscular strength” should concentrate on developing flexibility in addition to muscular strength and speed training, according to the study’s findings. This study discovered that children in each category who had high muscle strength did not have increased flexibility, suggesting that kindergarten sports should emphasize relaxation and stretching activities after school; in group B “average physical fitness,” physical fitness should be developed in an integrated manner; in group C “low agility,” low body strength should be developed comprehensively. Moreover, in this group, toddlers have relatively underdeveloped low-body muscles because they require muscular strength to rotate their bodies at high speeds [36] and have a relatively high BMI. Studies have shown that high-intensity physical activity in early childhood can have long-term effects by improving body composition and fitness, particularly on muscular strength [37]. The 3-year-old age group is characterized primarily by the natural, unconscious performance of various activities, which may emerge in numerous distinct categories of people. The reason is that average child growth and development patterns can be considered.

By balancing and maintaining the body's center of gravity perpendicular to the base of support, an individual can stabilize the activity of their muscles and the position of their joints [38]. The static balance is closely correlated with the growth of lower body muscles (tibialis anterior, piriformis, gastrocnemius) [39]. This study shows that around 4–5 years old, lower body muscle strength and speed of group A “balanced” are weak. Young children’s lower limb muscle strength and quickness should be improved by physical activity. Taller kids have a higher center of mass, more postural instability, and worse balance than other kids, which is consistent with the results of the current study [40]. Group B “muscular strength” should emphasize daily flexibility and stretching of tendons and ligaments, including the coordination of breathing and rhythm of movement when performing physical activities in strength and speed. According to considerable research, slim children have low body fat, little muscular mass, and insufficient muscle tissue, which causes them to lack the strength, speed, and explosive power of children of average weight [41], consistent with the current study’s clustering findings.

In the age group of 5–6 years old, group C “low physical fitness type” had low BMI and the lowest scores in seated forward bending, balance beam, 10-m shuttle run, 20-m run, tennis throw, and double-legged jumps. BMI is negatively correlated with physical fitness [42], which is the same as the clustering results of this study. The high BMI of group B “obese,” who did not perform well on the balance beam, double-legged jumps, seated forward bending, 10-m shuttle run, and 20-m run, led to these results owing to the increase in the thickness of the skin folds of the body, which affects the extension of ligaments and muscles of the trunk, lower back, and hip joints of the young children [43]. This is consistent with the findings of Łukasz Kryst et al. [44]. Studies have shown that the physical fitness status of children in the preschool period is not only closely related to the management of sports in kindergartens but also influenced by parents’ overprotection of their children and modern poor lifestyles [45–47]. Inside the garden, most kindergartens emphasize intellect, arts, and physical fitness. Most kindergarten teachers are women who are not highly motivated to organize physical activities [48, 49], and the way, frequency, and intensity of physical activities vary from garden to garden. Outside the park, parents also have different exercise requirements and lifestyles for their children, which may be related to the emergence of the various categories of physical fitness mentioned above.

The physical fitness of young children between the ages of 3 and 4 years, 4 and 5 years, and 5 and 6 years were closely associated with morphological indicators when combined with the clustered contribution analysis. Body morphology parameters are essential in the early years. The number of obese children is rising owing to the recent improvements in living conditions, and parents and child health professionals are growing increasing concern about the physical health of obese children [50]. BMI, a measurement of body fatness and thinness that reflects nutritional status and physical condition, can be calculated using height and weight [51]. Evidence suggests that nutrition, physical activity, and sedentary habits affect young children’s BMI and may persist into adulthood [52]. Early childhood and adolescence are crucial times for physical development. Physical fitness in preschoolers has been linked to low obesity or BMI levels, according to several cross-sectional studies [36, 53–56]. Other studies found that physical fitness in 4-year-olds is positively correlated with fat-free adiposity but negatively correlated with overall adiposity [36]. Obesity will occur if the BMI is too high. Similarly, young children may experience negative consequences if their weight is too low [57], and may even result in poor growth and development, which may interfere with their ability to live an everyday life [58]. BMI is the most commonly used surrogate for obesity in children and adolescents in research, clinical, and nursing care. However, BMI does not reflect obesity well across all BMI categories [59, 60]. Severe obesity requires a reassessment of specific metrics as a surrogate for obesity to be the most accurate [61], for example, blood pressure and cholesterol. Therefore, focusing on BMI indicators in young children is essential to understand physical fitness.

The four comprehensive evaluation factors in this study were identified by factor analysis as follows: muscle strength factor, body morphology factor, cardiovascular function factor, and physical fitness composite factor. Muscle strength is the key element and has the most significant overall impact. The development of cardiovascular, obesity, mental health, and bone health is closely correlated with muscle strength, which is a physical fitness factor that rises steadily with age [61–63]. Additionally, people with low muscle strength risk dying later in life [64]. Developing fundamental motor skills and physical growth are key contributors to increasing muscle strength [65]. Young children with strong muscles can participate in games such as tug of war and jumping, and strong muscles can carry out highly complex tasks [66]. Body shape indicators are determined to be crucial for physical fitness according to cluster analysis and cluster contribution ratio, consistent with factor 2 generated from factor analysis. This result suggests that body shape indicators are essential for physical fitness from an early age. Hypertension in children and adolescents has become a significant concern in recent years owing to increased blood pressure being connected to the obesity pandemic. However, cardiovascular disease is still one of the primary causes of morbidity and mortality in adults [67]. We should capture the muscle strength and body shape indicators while not neglecting the cardiovascular function and other indicators to increase overall strength and focus on developing the critical factors, to achieve the effect of pulling one hair to move the whole body [61, 68, 69].

### Strengths and limitations

#### Strengths

The current study shows that the physical health assessment of young children is only grouped according to age, but the problem of high variability of physical fitness between different stages of the same age has not been well solved. Cluster analysis and factor analysis are very advanced statistical methods, but few scholars have combined cluster analysis and factor analysis with physical fitness of preschool children. Using these two statistical methods, we can identify the physical characteristics of young children in the same age group and help them to develop individualised intervention plans. Factor analysis can also extract several key factors in the factors affecting young children's physical fitness, which not only helps young children grow up quickly and healthily, but also helps kindergartens develop physical activities to provide a scientific basis.

### Limitations

The present study is cross-sectional, and the extrapolation of findings may be constrained by the small sample size, geographic factors, economic factor, and lack of precise survey data on early-life variables, such as mothers' pre-pregnancy weight and weight before giving birth and early disease screening in young children. Future intervention training instruction for young children is possible if we undertake a longitudinal follow-up study on a fixed population.

## Conclusions

The present study is cross-sectional. The extrapolation of findings may be constrained by the small sample size and lack of precise survey data on early-life variables, such as mothers’ pre-pregnancy weight and weight before giving birth and early disease screening in young children. Future intervention training instruction for young children is possible if we undertake a longitudinal follow-up study on a fixed population. This study can draw the following conclusions: 1) Test indications differ substantially among young boys and girls. Women should focus on motor function and strength, while men on flexibility. Male group B “low physical fitness” should focus on strength, motor function, and balance, whereas male group A “high physical fitness” should focus on flexibility. Then, female group A “high physical fitness” should emphasize variety.2) For 3–4-year-olds, group A “muscular strength” should focus on flexibility, and group C “low agility” on motor function. 3) For 4–5-year-olds, group A “balanced” should focus on strength and motor function; 4) For 5–6-year-olds, group B “obese” should emphasize weight loss, and group C “low fitness” should emphasize strength, motor function, and flexibility; 5) Young children’s physical fitness depends on muscle strength, body shape, cardiovascular factors, and physical fitness composite.

### Supplementary Information

**Additional file 1.**

## Data Availability

The data analysed during the current study are available from the corresponding author on reasonable request.

## Abbreviation

BMI: Body Mass Index
